# Three-Dimensional Retinal Organoids Facilitate the Investigation of Retinal Ganglion Cell Development, Organization and Neurite Outgrowth from Human Pluripotent Stem Cells

**DOI:** 10.1038/s41598-018-32871-8

**Published:** 2018-09-28

**Authors:** Clarisse M. Fligor, Kirstin B. Langer, Akshayalakshmi Sridhar, Yuan Ren, Priya K. Shields, Michael C. Edler, Sarah K. Ohlemacher, Valentin M. Sluch, Donald J. Zack, Chi Zhang, Daniel M. Suter, Jason S. Meyer

**Affiliations:** 1 Department of Biology, Indiana University Purdue University Indianapolis, Indianapolis, IN 46202 USA; 2 Department of Biological Sciences, Purdue University, West Lafayette, IN 47907 USA; 3 Purdue Institute for Integrative Neuroscience, Purdue University, West Lafayette, IN 47907 USA; 4 Department of Molecular Biology and Genetics, Johns Hopkins University, Baltimore, MD 21287 USA; 5 Department of Ophthalmology, Wilmer Eye Institute, Johns Hopkins University, Baltimore, MD 21287 USA; 6 The Solomon H. Snyder Department of Neuroscience, Johns Hopkins University, Baltimore, MD 21287 USA; 7 Institute of Genetic Medicine, Johns Hopkins University, Baltimore, MD 21287 USA; 8 Department of Medical and Molecular Genetics, Indiana University, Indianapolis, IN 46202 USA; 9 Stark Neurosciences Research Institute, Indiana University, Indianapolis, IN 46202 USA; 10 Present Address: Department of Biological Structure, University of Washington, Seattle, WA 98195 USA

## Abstract

Retinal organoids are three-dimensional structures derived from human pluripotent stem cells (hPSCs) which recapitulate the spatial and temporal differentiation of the retina, serving as effective *in vitro* models of retinal development. However, a lack of emphasis has been placed upon the development and organization of retinal ganglion cells (RGCs) within retinal organoids. Thus, initial efforts were made to characterize RGC differentiation throughout early stages of organoid development, with a clearly defined RGC layer developing in a temporally-appropriate manner expressing a complement of RGC-associated markers. Beyond studies of RGC development, retinal organoids may also prove useful for cellular replacement in which extensive axonal outgrowth is necessary to reach post-synaptic targets. Organoid-derived RGCs could help to elucidate factors promoting axonal outgrowth, thereby identifying approaches to circumvent a formidable obstacle to RGC replacement. As such, additional efforts demonstrated significant enhancement of neurite outgrowth through modulation of both substrate composition and growth factor signaling. Additionally, organoid-derived RGCs exhibited diverse phenotypes, extending elaborate growth cones and expressing numerous guidance receptors. Collectively, these results establish retinal organoids as a valuable tool for studies of RGC development, and demonstrate the utility of organoid-derived RGCs as an effective platform to study factors influencing neurite outgrowth from organoid-derived RGCs.

## Introduction

Retinal ganglion cells (RGCs) play a critical role in the transmission of visual information between the eye and the brain, with many retinal degenerative diseases leading to the damage and loss of RGC axons^1–3^. As RGCs have a limited capacity for regeneration following damage^4,5^, previous efforts to restore RGC connections have been limited by numerous obstacles, including an inability to regrow long-distance connections. Additionally, at later stages of RGC degeneration following cell death, a need exists to replace the large number of cells that have been lost. Human pluripotent stem cells (hPSCs), including both embryonic and induced pluripotent stem cells, are attractive candidates for translational approaches, due to their ability to divide indefinitely as well as differentiate into any cell type in the body^6–8^, including those of the retina^9–16^.

Recent studies have demonstrated the ability to differentiate hPSCs into RGCs^17–21^, resulting in cells possessing appropriate morphological and functional properties. However, these RGCs were often derived in a stochastic manner, with cells lacking the organization typical of the retina, including the cell-to-cell interactions associated with retinogenesis. As such, their ability to serve as a model of retinal development is limited, as well as their utility for cell replacement therapies. More recently, studies have demonstrated the differentiation of hPSCs into optic cup-like retinal organoids, which allow for the generation of all cell types of the retina in a three-dimensional organized structure and provide access to some of the earliest events of retinogenesis that would otherwise be inaccessible to investigation^22–26^. However, these studies have focused on outer retinal cells such as photoreceptors, with a lack of emphasis upon the development of RGCs within retinal organoids. The differentiation of retinal organoids in a manner that closely mimics the spatial and temporal development of RGCs would provide a superior and more representative model of RGC development, facilitating applications of hPSC-derived RGCs for disease modeling, drug screening, as well as cell replacement.

Before the implementation of hPSC-derived RGCs for many of these applications, significant obstacles remain, including the ability to extend axons across long distances as well as the capacity to appropriately respond to extrinsic guidance cues to regulate this outgrowth. While animal models have provided a wealth of information about the mechanisms underlying RGC outgrowth^27–31^, little is known about how human RGCs respond to both intrinsic and extrinsic cues to regulate their neurite outgrowth. The differentiation of retinal organoids from hPSCs provides a population of RGCs that more faithfully recapitulates their spatial and temporal development within the retina and thus, may serve as a more effective *in vitro* model of RGC axonal outgrowth.

To this end, efforts were undertaken to examine the ability of hPSC-derived retinal organoids to serve as a reliable model of RGCs development, including their ability to extend lengthy neurites characteristic of these cells. RGCs were found to be the earliest cell type differentiated within retinal organoids, indicating their temporally-appropriate development, and expressed numerous characteristic markers. Additionally, the long distance outgrowth of neurites from hPSC-derived RGCs was analyzed, with this outgrowth regulated by extrinsic factors including both substrate composition as well as signaling via growth factors. Upon further analysis of extending neurites, F-actin-enriched growth cones were evident at their leading edge. Single cell transcriptomics confirmed that these hPSC-derived RGCs exhibited profound diversity, with varying patterns of expression of axon guidance receptors. Taken together, these results demonstrate the use of hPSC-derived retinal organoids as a powerful *in vitro* model of RGC development, with subsequent applications for studies of RGC outgrowth and guidance.

## Results

### Self-organization of RGCs within retinal organoids

**Figure 1:**
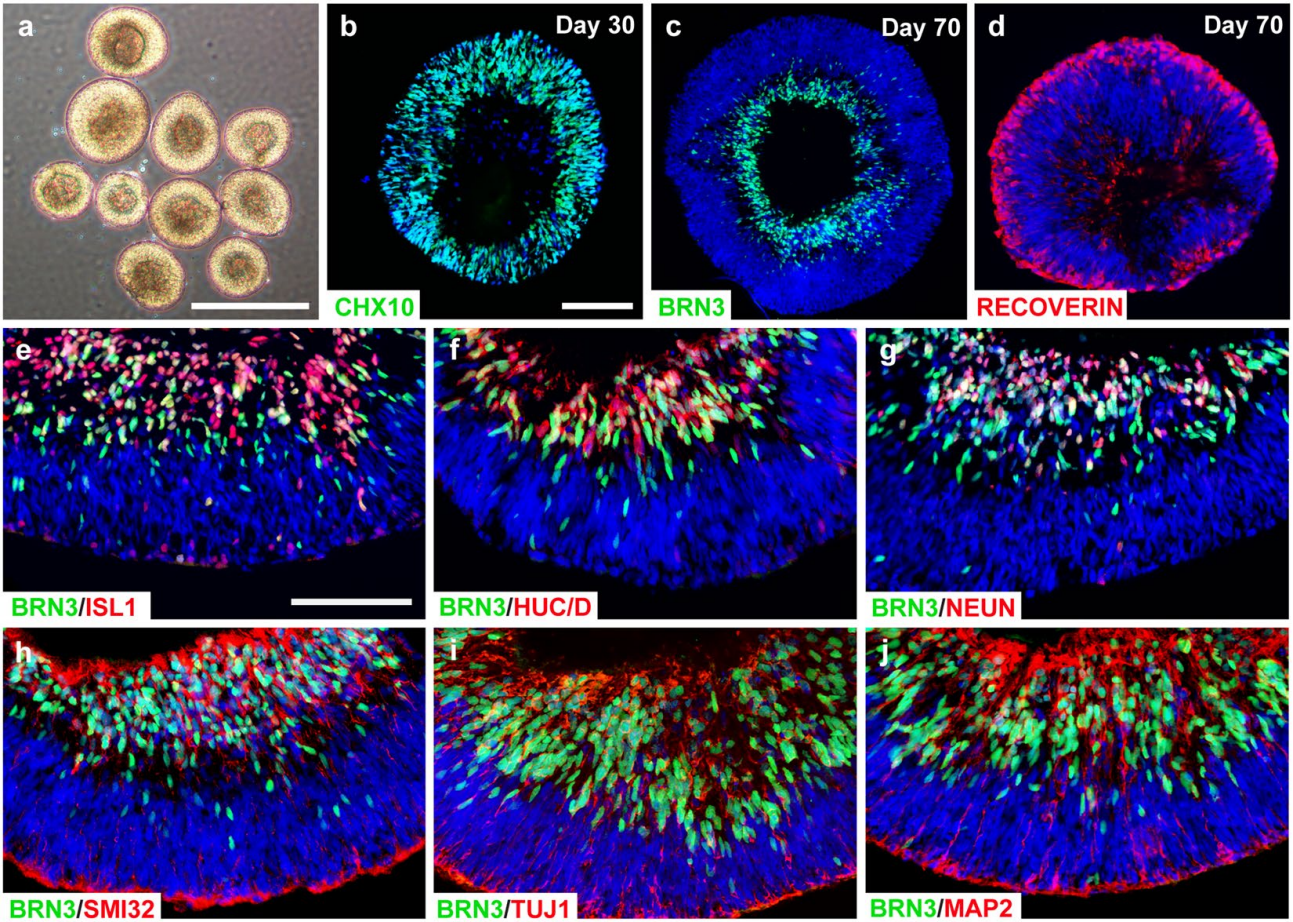
Organization of a distinct ganglion cell layer within retinal organoids. (**a**) Brightfield microscopy displayed the stratified morphology of early retinal organoids. (**b**) Within 30 days of differentiation, nearly all cells within retinal organoids expressed the retinal progenitor marker CHX10. (**c–d**) Within 70 days of differentiation, a presumptive ganglion cell layer expressing BRN3 occupied basal layers of retinal organoids, while Recoverin-positive photoreceptors resided in more apical layers. (**e**–**g**) RGCs that were found within the presumptive ganglion cell layer expressed characteristic RGC-associated proteins including BRN3, ISLET1, HuC/D, and NEUN. (**h**–**j**) BRN3-positive RGCs also co-expressed numerous characteristic cytoskeletal markers such as SMI32, TUJ1, MAP2. Scale bars equal 500μm for (**a**) and 100μm for (**b**–**j**). Scale bar in b applies to (**c**,**d**), scale bar in e applies to (**f**–**j**).

Retinal ganglion cells develop within a defined set of spatial and temporal parameters within the retina, with RGCs appearing as the first cell type to be specified within the innermost layers and identified by the expression of a variety of unique markers^32,33^. In parallel to the native events, initial efforts were focused upon the differentiation of retinal organoids in a manner that recapitulates both the spatial and temporal events of RGC development. Within 30 days of differentiation, retinal organoids displayed unique morphological and phenotypic characteristics, with a bright stratified layer toward the periphery indicative of their organized nature (Fig. 1a). At this stage of differentiation, retinal organoids uniformly expressed the retinal progenitor marker CHX10, analogous to the optic vesicle-stage of development (Fig. 1b). Upon further differentiation, RGCs self-organized within the basal layers of retinal organoids as identified by the expression of BRN3, with photoreceptors differentiating toward more peripheral regions, corresponding to the native location and stratification of layers within the retina (Fig. 1c,d). As a number of markers have been used to identify RGCs within the retina, a detailed characterization of RGCs within retinal organoids demonstrated the corresponding expression of BRN3 co-localized with other RGC markers within the inner layers of the three-dimensional structures (Fig. 1e–j). Thus, the differentiation of hPSC-derived retinal organoids allowed for the self-organization of RGCs into a defined layer in a manner mimicking the spatial organization of RGCs within the retina.

**Figure 2:**
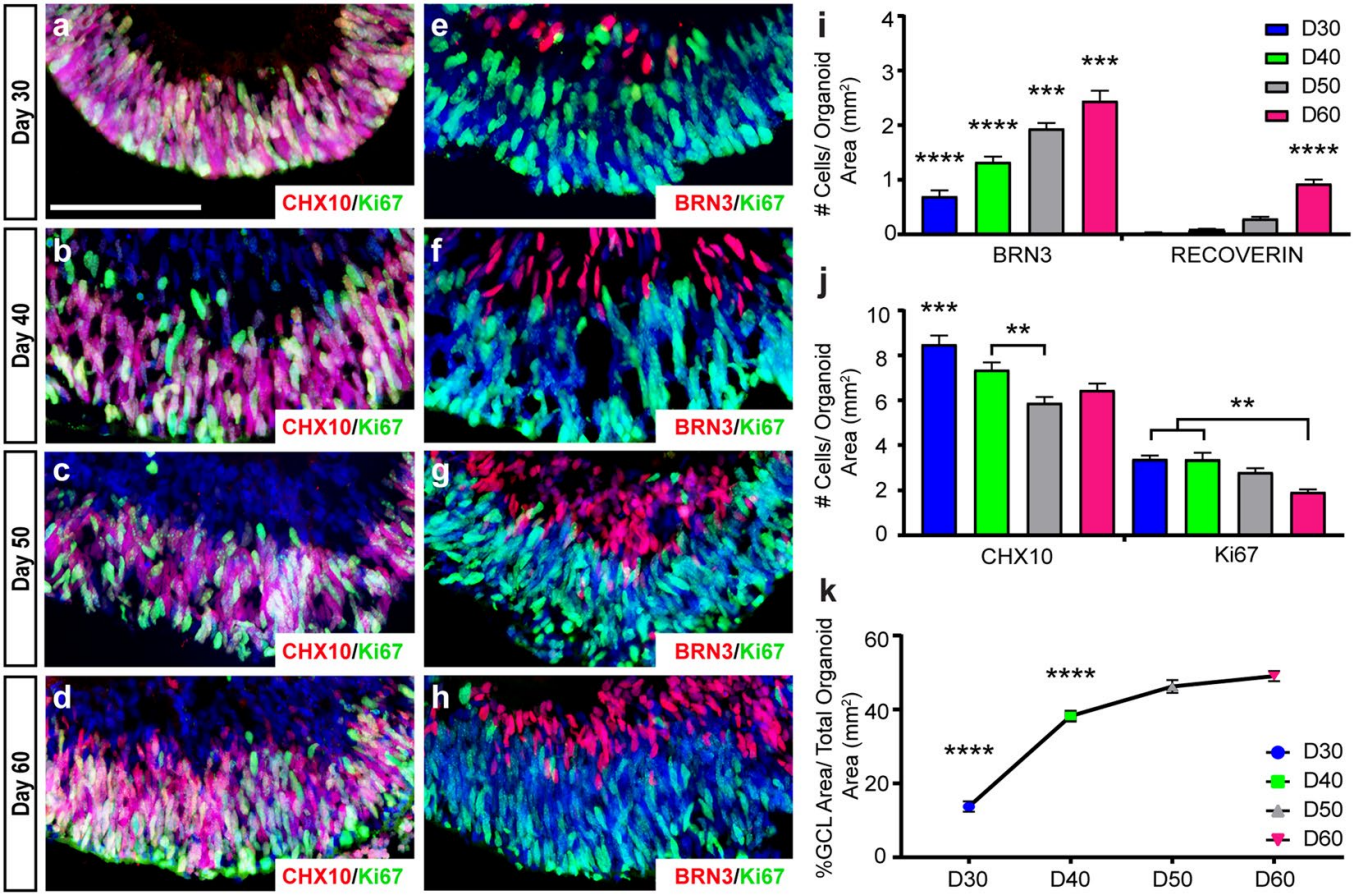
Spatial and temporal development of RGCs within retinal organoids. (**a–d**) The expression of retinal progenitor markers including CHX10 and Ki67 were widely expressed at early stages of organoid development, but became more restricted to outer layers by 60 days of differentiation. (**e–h**) The restriction of progenitor cells to the outer layers over time was associated with an increase in the expression of the RGC-associated marker BRN3 in the inner layers. (**i**) Quantification of immunostaining demonstrated a significant increase in BRN3 over time, followed by the delayed onset of expression of photoreceptor markers such as Recoverin. (**j**) Conversely, the number of CHX10- and Ki67-positive retinal progenitors significantly decreased over time. (**k**) Correlated with the advancement of RGC differentiation, the presumptive RGC layer area displayed a significant increase in its size over time relative to the size of the organoid. Error bars (n = 3) represent s.e.m. (**p < 0.01, ***p < 0.005, ****p < 0.001). Scale bars equal 100 μm.

Within the native retina, RGCs are also known to be the first cell type to be specified^32,33^. Thus, efforts focused upon the temporal development of RGCs within hPSC-derived organoids. Within 30 days of differentiation, retinal organoids exhibited widespread expression of retinal progenitor markers including CHX10 and Ki67, with the prevalence of these markers becoming more restricted to outer layers over time (Fig. 2a–d). Conversely, BRN3 expression identified few RGCs at 30 days of differentiation, with the appearance of RGCs within inner layers of retinal organoids becoming more abundant over time (Fig. 2e–h). Quantification of these results demonstrated that the onset of BRN3 expression occurred earlier than other retinal cell types such as photoreceptors, and corresponded with a decrease in the expression of progenitor markers (Fig. 2i,j). In parallel to the increased differentiation of RGCs within retinal organoids, the area of each organoid occupied by RGCs also increased over time (Fig. 2k). Thus, the differentiation of hPSC-derived retinal organoids also allowed for modeling of the temporal development of RGCs, in a manner similar to that observed within the bona fide retina.

### Identification of factors modulating RGC neurite outgrowth

As the projection neurons of the retina, RGCs must extend axons over long distances to reach postsynaptic targets^1–3^. Retinal organoids allow for the differentiation of RGCs in a spatial and temporal manner reflecting the development of the retina, providing a more physiologically-relevant model for studies of retinogenesis, as well as applications including disease modeling, drug screening, and cell replacement. Among the RGC-associated features that may be most important for many of these types of studies, the long-distance projection of neurites is a characteristic with important implications for developmental studies, disease modeling, as well as cell replacement. As such, the analysis of neurite outgrowth from retinal organoid-derived RGCs would facilitate and enhance a variety of translational applications.

**Figure 3:**
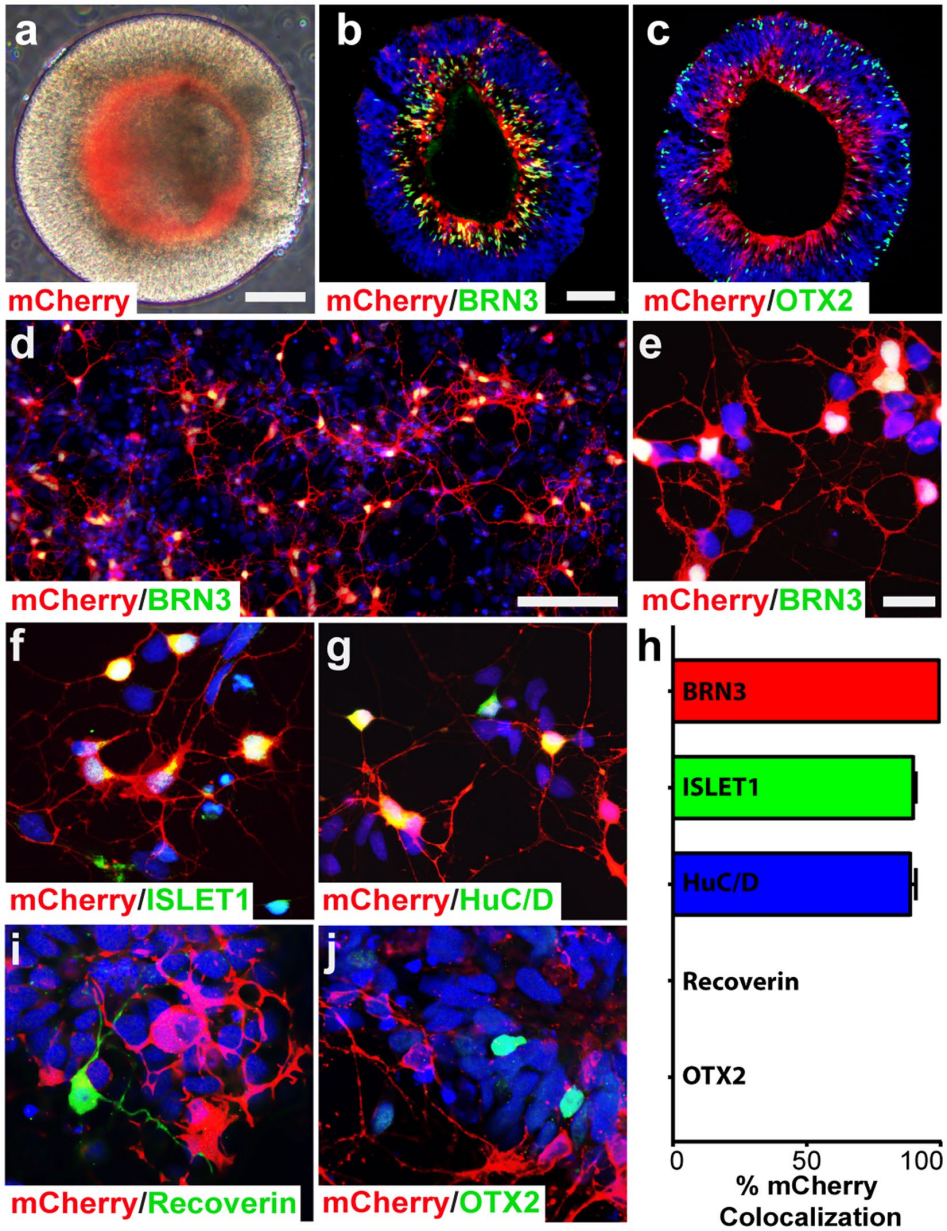
Identification of RGCs using a fluorescent reporter. (**a–c**) RGCs could be readily identified by mCherry expression observed in the inner layers of each organoid, defining the presumptive retinal ganglion cell layer. (**d–h**) Multiple RGC-associated markers such as BRN3, ISLET1, and HuC/D co-expressed with mCherry. (**h–j**) Conversely, mCherry did not co-localize with markers of other retinal cells such as Recoverin and OTX2. Error bars represent s.e.m. Scale bars: 100 μm (**a–c**), 200 μm (**d**), 20 μm (**e–g**, **i–j**).

As the derivation of RGCs within retinal organoids occurs along with the differentiation of other retinal cell types^22–26^, the definitive identification of RGC neurites apart from those of other retinal neurons is imperative. As such, a CRISPR engineered knock-in reporter cell line was utilized for these studies in which a red fluorescent reporter was expressed under the control of the retinal ganglion cell-associated gene BRN3B, with presumptive RGCs identified by the expression of either the BRN3B:mCherry or BRN3B:tdTomato reporter^19,34^ (Fig. 3, Supplementary Fig. S1). Upon differentiation of hPSCs into retinal organoids, expression of the BRN3B:mCherry reporter was specifically observed in the inner layers, defining a presumptive RGC layer (Fig. 3a), with mCherry-expression co-localized with BRN3 expression (Fig. 3b). The inner RGC layer appeared distinctly separate from the developing photoreceptor layer on the periphery (Fig. 3c). In order to better observe the morphological development of RGCs and ensure the specificity of the reporter, organoids were enzymatically dissociated and cells subsequently plated to allow for neurite outgrowth, with mCherry expression remaining strongly colocalized with multiple RGC markers (Fig. 3d–h). Additionally, the expression of markers indicative of other retinal lineages was absent from BRN3B:mCherry-positive RGCs (Fig. 3h–j). Thus, this reporter cell line provided an effective means of identifying RGCs apart from other cell types, including the neurites extending from these mCherry-expressing RGCs.

**Figure 4:**
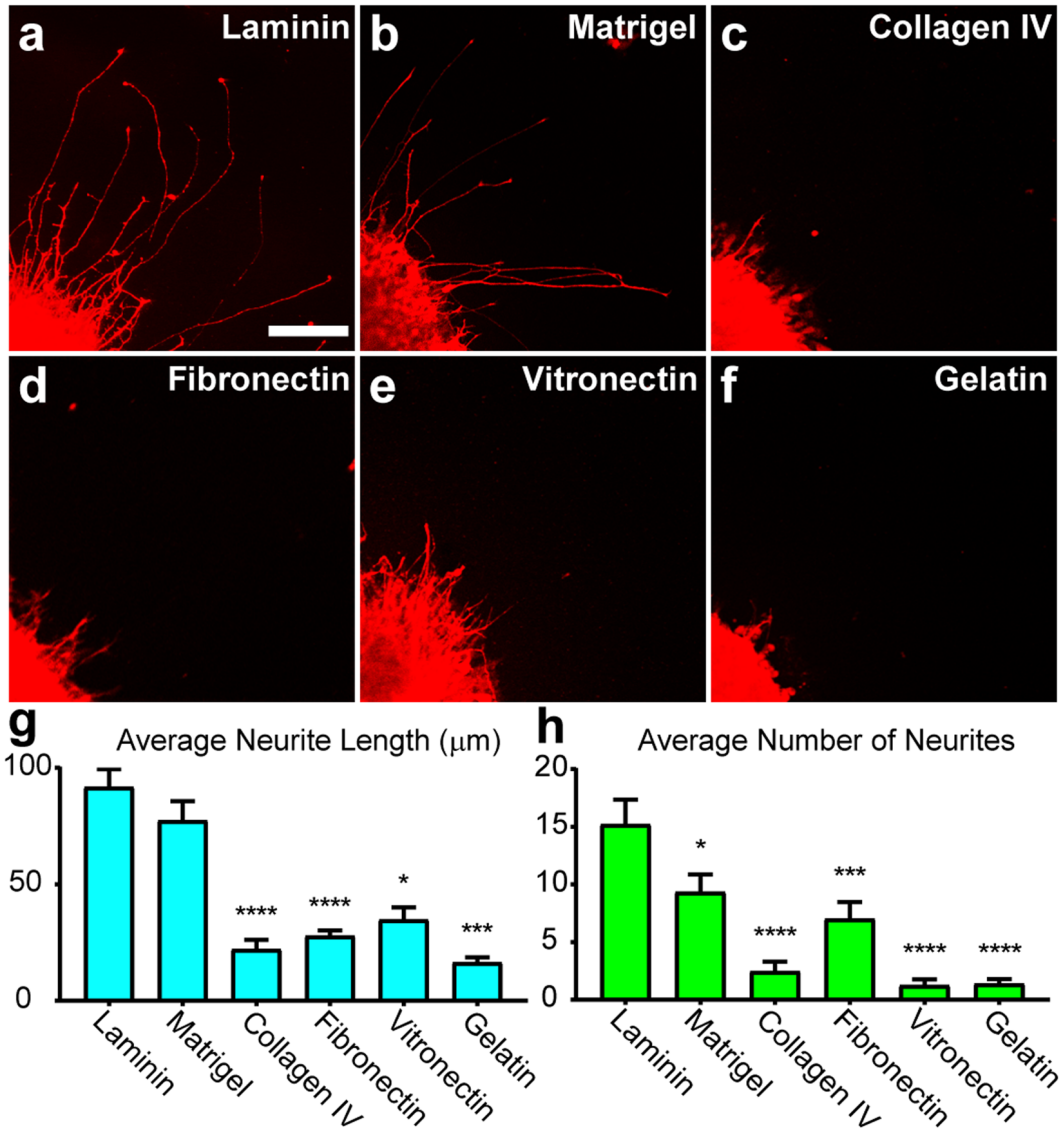
Substrate Modulation of RGC Neurite Outgrowth. (**a–f**) mCherry-positive RGCs were analyzed for neurite outgrowth on various substrates including laminin (n = 50), Matrigel (n = 51), collagen IV (n = 28), fibronectin (n = 39), vitronectin (n = 32) and gelatin (n = 16) where n is the number of aggregates analyzed. (**g**,**h**) Mean neurite length and mean number of neurites were calculated for each of the experimental conditions. Significant differences from laminin were determined by one-way ANOVA, *p < 0.05, ***p < 0.005, ****p < 0.001. Error bars represent s.e.m. Scale bar: 100 μm.

For the success of many translational applications of retinal organoid-derived RGCs, axons must extend over long distances to faithfully recapitulate their morphological features found *in vivo*. To accomplish this, RGCs must appropriately respond to extrinsic guidance cues that encourage and modulate long distance outgrowth. As such, determining how extrinsic factors regulate retinal organoid-derived RGC outgrowth would enhance efforts to develop models for disease modeling and cell replacement. As the extracellular matrix is known to be highly influential in the initial outgrowth of RGC neurites^35–40^, a variety of substrates commonly associated with the retina were tested for their effects upon retinal organoid-derived RGC neurite outgrowth (Fig. 4), with RGC neurites identified by the expression of mCherry. While RGCs were capable of some degree of growth in the presence of each substrate, results indicated that laminin and Matrigel were the most conducive for increased neurite length, while laminin allowed for significantly more neurites to extend compared to other substrates. Thus, laminin was utilized as the substrate for all subsequent experiments to optimize RGC neurite outgrowth. In addition to the extracellular matrix, the environment in which developing RGCs are found is also highly influential to their growth^28,41–46^. As an *in vitro* system with which to analyze neurite outgrowth, multiple culture media formulations were tested for their ability to influence neurite length as well as the number of neurites (Supplementary Fig. S2). Results indicated that RDM, NB-Sato, BrainPhys, and NIM were all comparable for average neurite length as well as the number of neurites extended, although the use of 10% FBS in DMEM significantly decreased both parameters.

**Figure 5:**
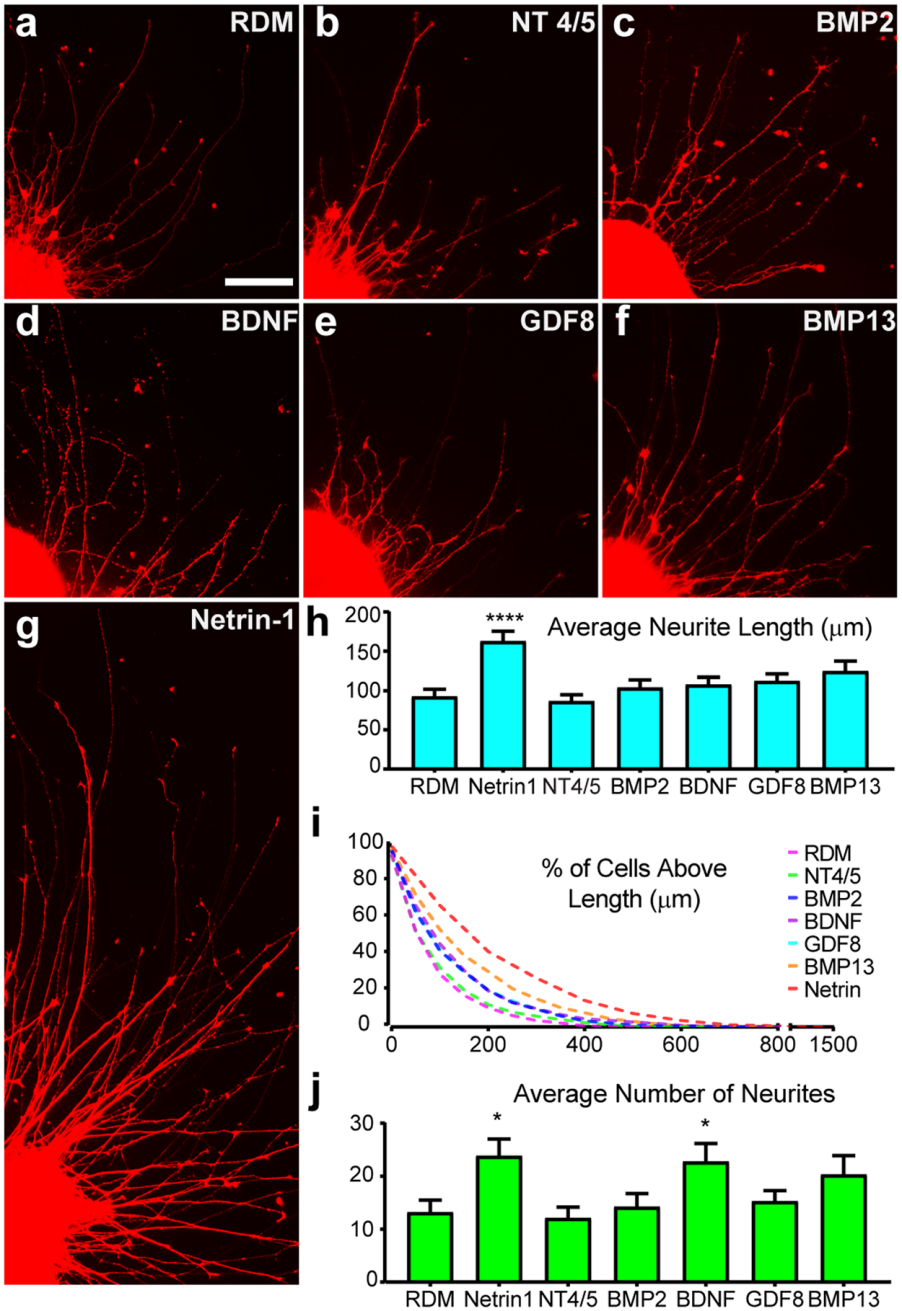
Modulation of Neurite Outgrowth by Soluble Factors. (**a–g**) mCherry positive RGCs were analyzed for neurite outgrowth with the addition of various growth factors including NT 4/5 (n = 36), BMP2 (n = 39), BDNF (n = 44), GDF8 (n = 55), BMP13 (n = 44) and Netrin-1 (n = 63) at a concentration of 50 ng/mL, where n is the number of aggregates analyzed. (**h**) Among the soluble factors tested, Netrin-1 was observed to significantly increase the average length of neurites. (**i**) All traced neurites were graphed as cumulative frequency above indicated length for each growth factor, with Netrin-1 producing neurites reaching lengths approaching 1.5 mm in 24 hours. (**j**) The number of neurites was also significantly increased in response to Netrin-1 and BDNF compared to RDM controls. Significance was determined by one-way ANOVA, *p < 0.05, ****p < 0.001. Error bars represent s.e.m. Scale bar: 100 μm.

The ability to guide and direct RGC axons to establish long distance connections is also known to depend on their responsiveness to a variety of growth factors and guidance cues^27,31,43,45,47,48^. Additionally, animal models have identified a number of growth factors which promote the survival and growth of RGCs^28,29,31,49,50^. As such, a panel of candidate growth factors was established and were individually tested for their ability to modulate neurite outgrowth from retinal organoid-derived RGCs (Fig. 5a–g). While neurite outgrowth was observed in control conditions as well as in the presence of all growth factors, Netrin-1 was found to significantly increase the average length of neurites extended (Fig. 5h), with some neurites reaching maximal lengths approaching 1.5 mm within the first 24 hours of growth (Fig. 5i). Additionally, retinal organoid-derived RGCs were able to extend significantly more neurites in the presence of both Netrin-1 as well as BDNF (Fig. 5j). Thus, these collective results establish an *in vitro* model with which to analyze neurite outgrowth from retinal organoid-derived RGCs, with enhanced neurite outgrowth facilitating the eventual use of retinal organoid-derived RGCs for studies of development, disease modeling and cell replacement.

### Neurite outgrowth and guidance receptor expression of RGCs

**Figure 6:**
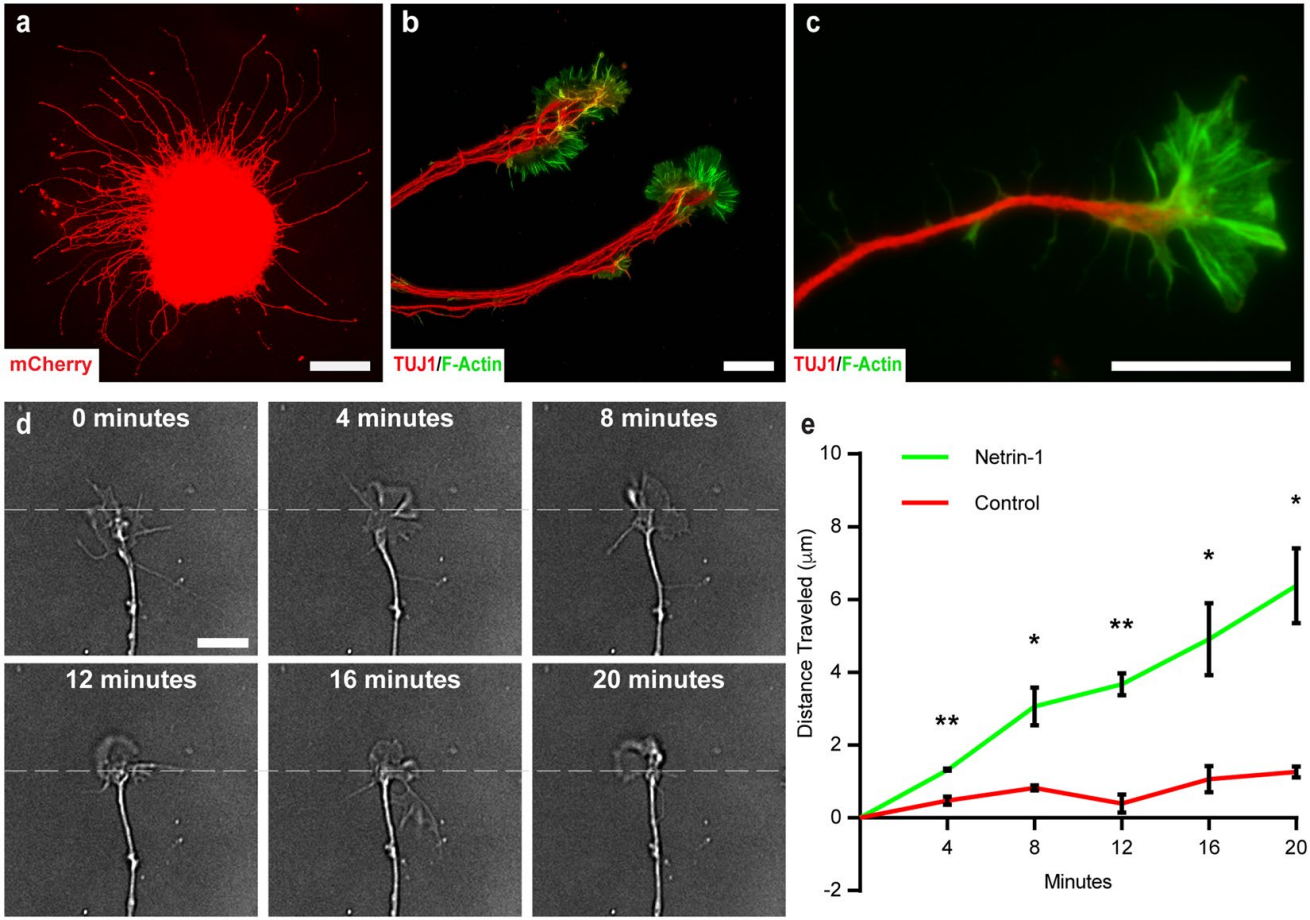
Identification and Dynamic Rearrangement of RGC Growth Cones (**a**) Cellular aggregates enriched for mCherry-positive RGCs extended numerous lengthy neurites in all directions after 24 hours in culture. (**b**) Neurites bundled together and displayed prominent growth cones at the leading edge of neurites. (**c**) Growth cones exhibited lamellipodia and numerous filopodia enriched for F-actin. (**d**) DIC time-lapse imaging revealed that these growth cones were also highly dynamic and motile over time, with apparent rearrangement and forward advancement of growth cones. (**e**) Growth cones exposed to Netrin-1 displayed a significant increase in forward growth compared to untreated controls. Significance was determined with multiple t-tests followed by the Holm-Sidak test. *p < 0.05, **p < 0.01. Error bars represent s.e.m. Scale bars: 150 μm (**a**), 25 μm (**b–d**).

Axons of RGCs navigate their way through the brain via growth cones at their leading edge, which respond to long and short range chemoattractants and chemorepellents^47,48,51,52^. Similarly, the outgrowth of neurites from retinal organoid-derived RGCs would be regulated by growth cones detecting changes in the environment. To examine these properties, retinal organoid-derived RGCs were analyzed for their ability to develop growth cone structures at the leading edge of neurites. Within 24 hours following plating, aggregates of retinal organoid-derived RGCs extended numerous neurites in all directions (Fig. 6a). Neurites could be seen bundling together to mimic the fasciculation of the optic nerve during development (Fig. 6b). Growth cones with F-actin-enriched lamellipodia and filopodia were identified at the leading edge of each neurite (Fig. 6b,c). Live cell imaging demonstrated that growth cones were highly motile and dynamic as seen by the continuous movement of filopodia and lamellipodia (Fig. 6d, Supplementary Movie 1). Interestingly, short term exposure of the same growth cones to Netrin-1 significantly increased the forward extension of RGC growth cones (Fig. 6e, Supplementary Movie 2).

**Figure 7:**
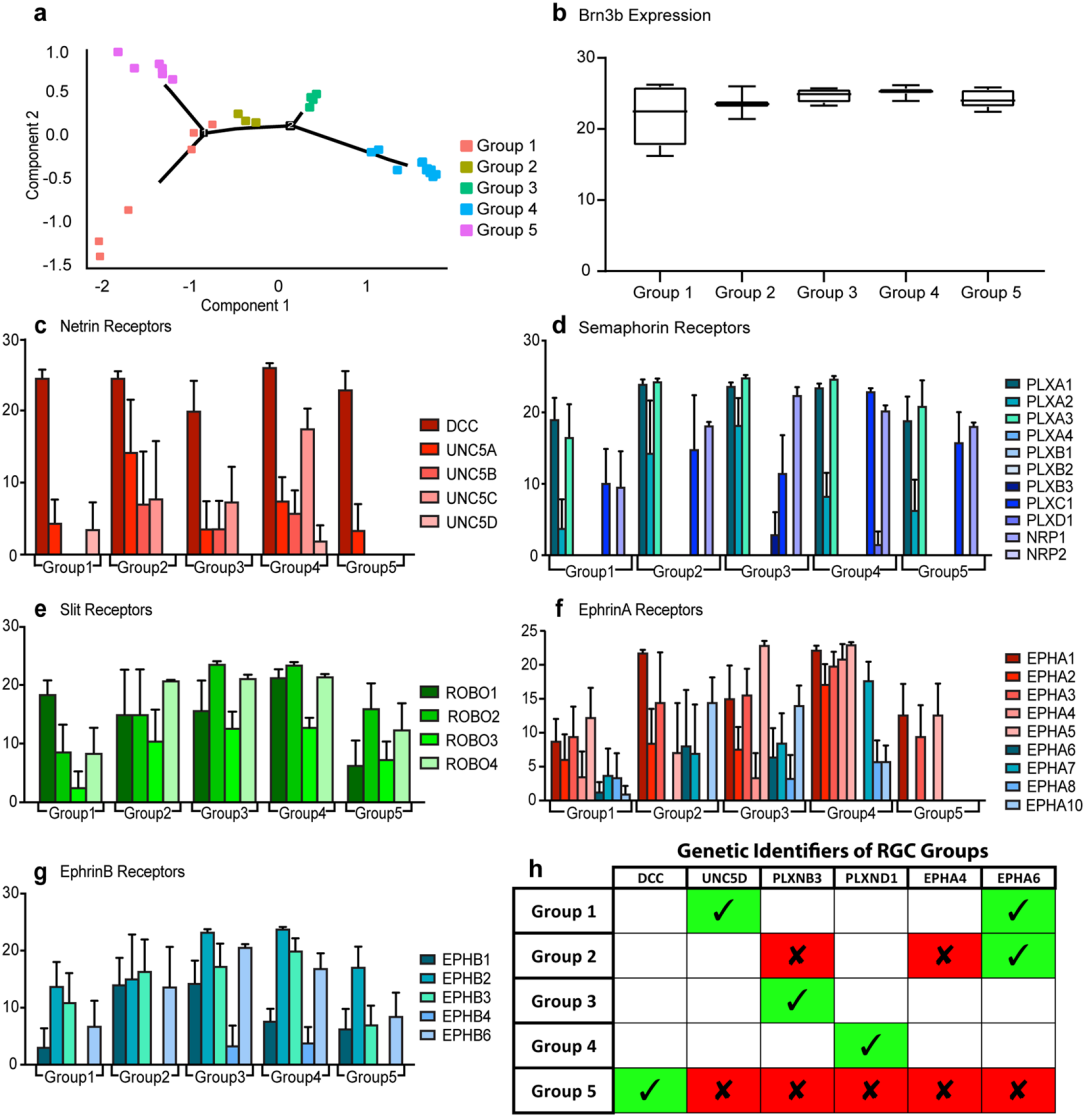
Diversity of Guidance Receptor Gene Expression in retinal organoid-derived RGCs. (**a**) Based on the expression of guidance receptor genes, individual RGCs were analyzed and grouped into five profiles based on gene expression (n = 33). (**b**) Each of the five groups displayed similar levels of the RGC marker BRN3b. (**c–g**) Relative gene expression of guidance receptor genes is shown for each of the five groups of RGCs. Gene expression levels are indicated on the Y-axes. Error bars represent s.e.m. (**h**) Of the 34 guidance receptor genes analyzed, unique expression profiles were generated for each group based on the expression of just 6 genes. Green boxes indicate genes whose expression is required for definitive identification of an RGC group, while red boxes indicate genes whose expression must be absent for definitive identification of that group. Unfilled boxes indicate that the presence or absence of the indicated gene is not a requirement for identification of that particular RGC group.

In order to better identify growth-cone associated guidance receptors for chemoattractant and chemorepellent molecules expressed specifically within retinal organoid-derived RGCs, the transcriptional profiles of individual RGCs were analyzed. Cells were FACS-sorted for their BRN3B:tdTomato expression to yield an enriched population of RGCs and single cell qRT-PCR analyses were performed to determine which receptors were expressed in these cells. Individual cells were screened for expression of multiple guidance receptor genes, and individual cells were clustered into five distinct groups based on similar gene expression profiles (Fig. 7a). BRN3B was found to be expressed similarly by all groups (Fig. 7b), however, varying expression levels of guidance receptor genes were observed between groups (Fig. 7c–g). Thus, while the presence of growth cones was universal among all RGC neurites, the composition of guidance receptors among these cells was heterogeneous, suggesting significant diversity within the broader retinal organoid-derived RGC population. The expression of these guidance receptor genes was then further analyzed to develop a unique signature profile for each of the RGC groups, with the expression of only 6 genes allowing for their definitive identification (Fig. 7h).

## Discussion

The results presented here demonstrate the ability to derive a primitive retinal ganglion cell layer within hPSC-derived retinal organoids in a manner that closely recapitulates the spatial and temporal patterning of RGC development. The development of retinal organoids with a defined presumptive RGC layer provides a more physiologically relevant model of retinogenesis, facilitating their use as *in vitro* models of RGC generation and organization, as well as providing a reliable platform to study hallmark characteristics of RGCs such as long distance axonal outgrowth and guidance. These latter characteristics were analyzed using retinal organoid-derived RGCs, with results demonstrating the modulation of RGC neurite outgrowth in response to a variety of signaling cues. The enhanced differentiation and three-dimensional organization of retinal organoid-derived RGCs provides a platform for studies of RGC development as well as disease modeling, and may facilitate the eventual application of hPSC-derived RGCs for cell replacement.

Compared to traditional stochastic methods of differentiation, retinal organoids are more similar to primary tissue in their cellular arrangement and stratification, with populations of progenitors giving rise to multiple differentiated cell types at frequencies similar to native tissue^22–26,53^. In the current study, RGCs were the first cell type to be specified, with the organization of these cells in the inner layers of retinal organoids reminiscent of the ganglion cell layer in the human retina. The onset of ganglion cell development, characterized by the expression of multiple RGC-associated markers, correlated with a decrease in retinal progenitor cells within the innermost layer of retinal organoids. In contrast, as time progressed retinal progenitor populations were maintained in outer retinal layers where later-born cell types, such as photoreceptors had yet to be specified. Further differentiation of these retinal organoids led to a progressive reduction in retinal progenitor populations, associated with a significant increase in the area of the presumptive ganglion cell layer as well as the onset of photoreceptor cells in outer layers. As such, differentiation of RGCs within retinal organoids mimicked the developmental timing and organization of the retina by allowing cells to self-organize into laminated structures, offering an advantage over traditional stochastic methods of RGC differentiation by providing a more physiologically-relevant, three-dimensional organization of retinal cells. At the same time, retinal organoids retained many of the advantages of *in vitro* systems in that they were grown within an environment that could be readily manipulated, eliminating confounding influence from the external environment^53^.

As projection neurons of the retina, RGCs extend long axons to reach postsynaptic targets^51,54,55^. The effective application of retinal organoid-derived RGCs would similarly require these cells to extend long neurites for proper disease modeling and drug screening, as well as their eventual use for cellular replacement. Previous studies have demonstrated the importance of the extracellular matrix as well as guidance cues in the local microenvironment in influencing the outgrowth and directionality of RGC axons, both during development as well as regeneration following optic nerve damage^27,30,43,48,52,56,57^. As the external environment around RGCs is highly influential to their development and axonal outgrowth, factors influencing these processes were tested, with laminin found to significantly enhance RGC neurite outgrowth. Interestingly, as laminin is known to be a primary component of the extracellular matrix in the nerve fiber layer^35,37,42,58^, retinal organoid-derived RGCs exhibited a physiologically-relevant preference for this substrate. Additionally, while few differences were observed between varying culture media formulations, basal media still supported outgrowth of neurites within 24 hours. Interestingly, a number of factors that have been previously demonstrated to modulate RGC neurite outgrowth did not elicit significant changes in our experiments. While the precise reason(s) for these differences are unclear, it is important to note the species differences between these studies, with many of these previous results obtained in rodent cells. Additionally, the developmental age of the RGCs could affect their responsiveness to certain factors, as RGCs are known to change their responsiveness during the course of their maturation. However, outgrowth was in fact found to be further enhanced by the addition of Netrin-1, resulting in a significant increase in the number of neurites extended as well as the average length of neurites. Importantly, Netrin-1 is known to be expressed by astrocytes in the optic nerve head, functioning to help guide RGC axons out of the eye and into the optic nerve^30,42,59^. Thus, the ability of retinal organoid-derived RGCs to respond to soluble and substrate-bound signals, particularly Netrin-1 and laminin, not only enables their use as an *in vitro* model of retinogenesis, but also reflects the developmental stage of these cells reminiscent of early RGC axonal outgrowth. The composition of influential growth factors and extracellular matrix components are known to vary, however, once RGC axons enter the optic nerve^38,42,60^. In the future, it will be important to determine how these extracellular matrices as well as soluble growth factors may differentially regulate the outgrowth of neurites from hPSC-derived RGCs at varying developmental stages.

During the development of RGCs, axons navigate across significant distances via growth cones that respond to multiple guidance cues, reducing the risk of error and leading to the intricate and accurate wiring of the visual pathway^47,48,51,52^. The data presented within the current study represents the most systematic analysis of retinal organoid-derived RGC growth cones to date, with RGC growth cones exhibiting distinctive F-actin organization in the peripheral domain. Interestingly, hPSC-derived RGC growth cones exhibited dynamic rearrangement of their actin cytoskeleton as they advanced forward. Moreover, when the same growth cones were exposed to Netrin-1, forward growth rate increased significantly. As Netrin-1 response is mediated by receptors such as DCC and UNC, the presence of growth cones was associated with the expression of a number of axon guidance receptor genes that are known to allow for growth cone navigation^48,51,59^.

Furthermore, a systematic analysis of RGCs by single cell qRT-PCR directly identified receptors specifically expressed by individual RGCs. Although expression of guidance receptors has been shown in animal models, expression in human retinal organoid-derived RGCs had yet to be determined. Analysis of single cell qRT-PCR data via t-SNE revealed a spatial clustering of these cells into five defined groups based upon their gene expression profiles. Interestingly, the expression of only 6 out of the 34 guidance receptor genes could be utilized to identify these specific groups of RGCs, with the expression of some guidance receptors unique to individual groups. While the exact significance of these groups remains unclear, each group may represent an important developmental or functional category of RGC. Based on the differential expression of guidance receptors, these five groups of RGCs may reflect differences in their post-synaptic targets, as various regions would require different combinations of guidance cues to appropriately direct RGC axons^61^. For example, all groups express receptors influential in guiding RGCs out of the eye, yet vary in levels of other retino-tectal patterning genes influential in determining post-synaptic targets of RGCs. Alternatively, these groups may correlate with different subtypes of RGCs, each of which are known to possess different morphological, molecular, and physiological properties^62–65^. Furthermore, these cells may simply be at various stages of development, with some cells slightly more advanced in this process compared to others. Finally, the most likely explanation is the five groups represent a combination of all factors, leading to a degree of heterogeneity within the RGC population, as previously demonstrated in animal models^66^. Nevertheless, results demonstrate which guidance receptors were expressed in retinal organoid-derived RGCs, supporting the concept that these cells represent a heterogeneous population capable of responding to various intrinsic and extrinsic signals that may guide them to their respective targets within the brain.

As the primary connection between the eye and the brain, RGCs serve a critical function in visual transduction pathways. Numerous degenerative disorders adversely affect the ganglion cells of the retina, leading to their degeneration and eventual loss^1–3^. While early intervention strategies are often focused upon the neuroprotection of these cells, a critical need exists for cellular replacement strategies at later stages of degenerative diseases once a large number of RGCs has been lost^67–70^. However, in order for successful replacement of RGCs to occur, axons will have to extend across significant distances to reach appropriate post-synaptic targets^51,55,71^. Additionally, once this axonal pathfinding is accomplished, RGC axons must form functional synaptic connections^54^. Thus, significant obstacles remain before hPSC-derived RGCs can be implemented for cell replacement. The demonstration of extensive neurite outgrowth from retinal organoid-derived RGCs provides a powerful approach for gaining a greater understanding of factors influencing the ability of hPSC-derived RGCs to be used for cell replacement applications. As such, data presented within this study represents an important step toward the eventual application of hPSC-derived RGCs for cell replacement purposes.

While the current results outline the use of retinal organoid-derived RGCs for studies of retinal development and neurite outgrowth, opportunities also exist to study factors that limit this growth. Optic neuropathies constitute a group of degenerative diseases which target RGCs, leading to loss of vision and blindness^72–76^. As such, the study of optic neuropathies with hPSCs would be greatly facilitated by the development of retinal organoids that effectively mimic the development and degeneration of the retinal ganglion cell layer. Moreover, the use of an *in vitro* model of RGC neurite outgrowth, particularly when utilizing hPSC-derived RGCs from a glaucomatous source, facilitates future studies in which the precise mechanisms underlying impaired outgrowth of glaucomatous RGCs can be analyzed. Furthermore, these future approaches can also enable the screening of compounds that encourage enhanced outgrowth from these cells, with important translational implications for the regeneration of diseased RGCs.

## Materials and Methods

### Maintenance and expansion of hPSCs

Four different lines of hPSCs were utilized in this study, including those with (A81-H7^19^ and E4-H7^34^) and without (H9^8^ and miPS2^14^) an RGC-specific fluorescent reporter. hPSCs were initially maintained in an undifferentiated state as previously described^77^. Briefly, cells were maintained in mTeSR1 medium on a Matrigel substrate. Upon reaching approximately 70% confluency, cells were mechanically passaged with dispase (2 mg/ml) and split at a ratio of 1:6, with passaging of cells occurring every 4–5 days.

### Differentiation of retinal organoids from hPSCs

hPSCs were differentiated in a stepwise manner to a retinal lineage following established protocols^77^. Briefly, hPSCs were lifted from the Matrigel-coated wells using dispase (2 mg/ml) and were kept in suspension as embryoid bodies (EBs). The EBs were gradually transitioned to a chemically defined neural induction medium (NIM), which consisted of DMEM/F12 (1:1), N2 supplement, MEM non-essential amino acids, heparin (2 µg/ml) and PSA. After seven days, the EBs were plated with 10% FBS in NIM to allow for adhesion. The following day, FBS was removed and cells were maintained in NIM, with media changed every other day. After 16 days of differentiation, cell aggregates were mechanically lifted and kept in suspension in Retinal Differentiation Medium (RDM), which consisted of DMEM/F12 (3:1), B27 supplement, MEM non-essential amino acids, and PSA. Retinal organoids containing presumptive RGCs were maintained in this medium until experimental time points indicated.

### Modulation of RGC neurite outgrowth

Retinal organoids were identified morphologically at a total of 40 days of differentiation as previously described^77^ and chopped to a uniform size of approximately 200 μm using a McIlwain tissue chopper (Stoelting). Cell aggregates were individually plated onto coverslips coated with poly-ornithine and an additional substrate as indicated experimentally. After 24 hours to allow for adhesion and neurite outgrowth, cells were fixed and analyzed by immunocytochemistry. For substrate experiments, coverslips were coated with the one of the following substrates for 4 hours following manufacturer’s instructions: laminin (20 µg/mL; Life Technologies), Matrigel (Corning), collagen IV (10 µg/mL, Stem Cell Technologies), fibronectin (1 µg/mL; Stem Cell Technologies), vitronectin (10 µg/mL; Stem Cell Technologies), or 0.1% gelatin (Sigma-Aldrich) solution. Substrates were diluted in DMEM, except for Collagen which was diluted in acetic acid and gelatin which was diluted in water. Samples were analyzed from seven individual experiments for a total N = 216 cellular aggregates. For testing the effects of culture media formulations on neurite outgrowth, laminin-coated coverslips were placed in one of the following medias; RDM^77^, NIM^77^, BrainPhys (StemCell Technologies)^78^, NB-Sato^46^, or 10% FBS in DMEM:F12. Seven experimental groups of cells were analyzed for a total N = 252. Finally, to test the effects of candidate growth factors upon neurite outgrowth, growth factors including NT4/5, BMP2, BMP13, GDF8, BDNF, or Netrin-1 were reconstituted in DMEM and added individually to laminin-coated coverslips in RDM at a final concentration of 50 ng/mL. Samples for growth factor analysis were obtained from four individual experimental groups of cells for a total N = 328.

To better visualize individual RGCs, retinal organoids were dissociated to single cells after 40 days of differentiation using Accutase for 20 minutes at 37 °C. The cell suspension was then plated onto poly-D-ornithine and laminin-coated coverslips at a concentration of 50,000 cells and maintained for up to an additional 40 days, for a total of 80 days of differentiation to allow for the differentiation of multiple retinal cell types.

### Immunocytochemistry and Imaging

For cryostat sectioning, retinal organoids were fixed with 4% paraformaldehyde, washed 3x in PBS, and then equilibrated in a 20% and then 30% sucrose solution overnight at 4 °C. Once reaching equilibrium, organoids were embedded in OCT and frozen on dry ice and sections were cut at 11 µm thickness. Similarly, RGCs grown on coverslips were fixed in 4% paraformaldehyde and washed 3x in PBS before staining.

Immunocytochemical staining of samples was performed as previously described^15,16^. Briefly, permeabilization was performed in 0.2% Triton X-100 for 10 minutes and samples were then blocked in 10% donkey serum for one hour at room temperature. Primary antibodies were diluted as indicated (Table S1) in 0.1% Triton X-100 and 5% donkey serum and applied overnight at 4 °C. The following day, samples were washed in PBS and blocked with 10% donkey serum for 10 minutes. Secondary antibodies were diluted 1:1000 in 0.1% Triton X-100 and 5% donkey serum and applied for one hour at room temperature. Finally, cells were washed with PBS and mounted onto slides for imaging.

For growth cone analyses, samples were fixed in 3.7% formaldehyde for 25 minutes at room temperature and permeabilized with 0.1% Triton X-100 for 10 minutes. For a blocking agent, 5% BSA was applied for 20 minutes. Primary antibodies (Table S1) and/or Phalloidin (Life Technologies, Cat. # A12379, 1:100) were prepared in 5% BSA and added to samples for 30 minutes at room temperature. Following 3 washes in PBS, secondary antibodies were also prepared in 5% BSA and added to samples for 30 minutes at room temperature. All samples were imaged with a Leica DM5500 fluorescence microscope.

Time-lapse imaging of growth cone dynamics was acquired on a Nikon TE2000 Eclipse inverted microscope using differential interference contrast (DIC) optics and a 60x objective with additional 1.5x magnification. Images were acquired with an Andor iXon Ultra 888 EM CCD camera at 10 second intervals. For control conditions, cells were plated in their respective growth medium on glass bottom petri dishes for time-lapse imaging. Following 20 minutes of imaging, 50 ng/mL of Netrin-1 was bath applied and the same growth cones were imaged for an additional 20 minutes.

### Quantification and statistical analysis

The number of cells expressing unique retinal markers was quantified in cryostat sections of retinal organoids at indicated timepoints. Multiple biological replicates were obtained at each time point (n = 3) and Image-J was used to quantify the expression of each marker as indicated in results. One-Way ANOVA statistical analyses at 95% confidence (post hoc Tukey) was performed, excluding outliers, to determine significant differences in cell counts over time. Statistical significances were determined based on a *p* value less than 0.05.

To analyze retinal organoid-derived RGCs, mCherry- or tdTomato-positive RGCs were quantified, and the co-expression of these reporters with RGC or other retinal cell type markers was quantified using the Image-J cell counter. Four distinct regions of at least three coverslips were imaged and quantified, with these experiments repeated with at least three different groups of cells. The percentage of mCherry-positive cells colocalizing with retinal cell type markers and the standard error of the mean was quantified with GraphPad Prism software.

In order to analyze and quantify the effects of experimental conditions upon neurite outgrowth, RGC neurites were identified by mCherry-expression and neurites were traced with a semi-automatic ImageJ plug-in, NeuronJ. The mean length of RGC neurites, as well as the mean number of neurites, were calculated along with the standard error of the mean. Grubb’s test was used to remove outliers with an alpha of 0.05. One-way ANOVA followed by Tukey’s post hoc or student’s two-tailed t-test using the Holm-Sidak method determined significance between samples, with *p* values less than 0.05 considered significant. Statistical analyses were performed using Graphpad Prism software.

### Single cell qRT-PCR analyses

For single cell qRT-PCR analyses, BRN3:tdTomato-expressing retinal organoids were dissociated to a single cell suspension using Accutase for 20 minutes at 37 °C. Cells were resuspended in 0.1% FBS in PBS and run through a cell strainer to yield a single-cell suspension. Single cells were sorted (BD SORP Aria) for TdTomato-positive cells, with viability of cells assessed by Propidium Iodide to ensure the collection of live, tdTomato-expressing cells. Resultant cells were loaded into the integrated fluidic circuit (Fluidigm) to isolate single cells into individual chambers. Cells were then lysed and cDNA was generated for qPCR analysis using the Biomark HD system (Fluidigm).

For qRT-PCR results, data from individual cells was analyzed and gene expression was defined with scores $${\rm{S}}=\{\begin{array}{c}40-CT\,value,\,if\,CT\le 40\\ 0,\,if\,CT > 40\end{array}$$ to reflect the gene’s expression levels in our qRT-PCR data. Higher values of S suggested higher gene expression levels. Of all cells analyzed, those without BRN3B expression were excluded from gene expression analysis. S values of all the analyzed genes were used to build a cell trajectory to reflect the distance of cells with respect to their gene expression profiles. Cell trajectory analysis was conducted by using the “Monocle” package in R under negative binomial assumption of S. Specially, t-SNE was first applied to reduce the data dimension. The top three components were significant and selected for trajectory construction.

## Electronic supplementary material

Supplementary Information

Supplementary Movie 1

Supplementary Movie 2
