# Supplementary material for: Three-Dimensional Retinal Organoids Facilitate the Investigation of Retinal Ganglion Cell Development, Organization and Neurite Outgrowth from Human Pluripotent Stem Cells
Source: Sci Rep. 2018 Sep 28;8:14520. doi: 10.1038/s41598-018-32871-8 (PMC6162218; doi:10.1038/s41598-018-32871-8)
Supplement: Supplementary file 1 — Supplementary Information [file 41598_2018_32871_MOESM1_ESM.pdf]

Three-Dimensional Retinal Organoids Facilitate the Investigation of Retinal Ganglion Cell Development, Organization and Neurite Outgrowth from Human Pluripotent Stem Cells

Clarisse M. Fligor[1], Kirstin B. Langer[1], Akshayalakshmi Sridhar[1¤], Yuan Ren[2,3], Priya K. Shields[1], Michael C. Edler[1], Sarah K. Ohlemacher[1], Valentin M. Sluch[4], Donald J. Zack[4-7], Chi Zhang[8], Daniel M. Suter[2,3], and Jason S. Meyer*[1, 8, 9]

Supplementary Table S1. Primary antibodies used for immunocytochemistry.

Antibody	Species	Source	Catalog Number	Dilution
BRN3	Goat	Santa Cruz Biotechnology	SC-6026	1:200
CHX10	Goat	Santa Cruz Biotechnology	SC-21690	1:200
HuC/D	Mouse	Molecular Probes	A-21271	1:200
ISLET1	Mouse	DSHB	40.2D6	1:200
Ki-67	Mouse	BD Biosciences	550609	1:500
MAP2	Rabbit	Santa Cruz Biotechnology	SC-20172	1:200
mCHERRY	Rabbit	Invitrogen	PA5-34974	1:500
NeuN	Mouse	Millipore	MAP377	1:100
OTX2	Goat	R&D Systems	AF1979	1:2000
RECOVERIN	Rabbit	Millipore	AB5585	1:2000
SMI32	Mouse	Calbiochem	CBL171	1:100
TUJ1	Mouse	Sigma	T8660	1:800

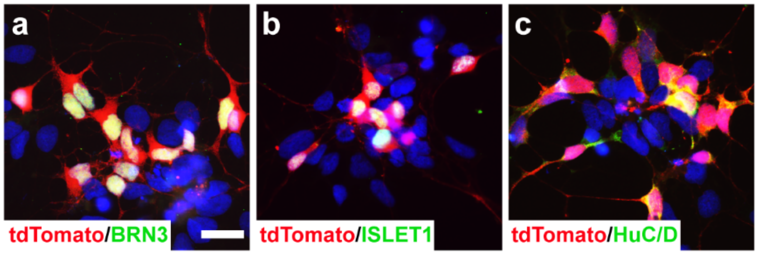

Supplementary Figure S1. Identification of RGCs using a BRN3B:tdTomato reporter. tdTomato-expressing presumptive RGCs co-expressed characteristic markers including BRN3, ISLET1, and HuC/D. Scale bar: 20 μm.

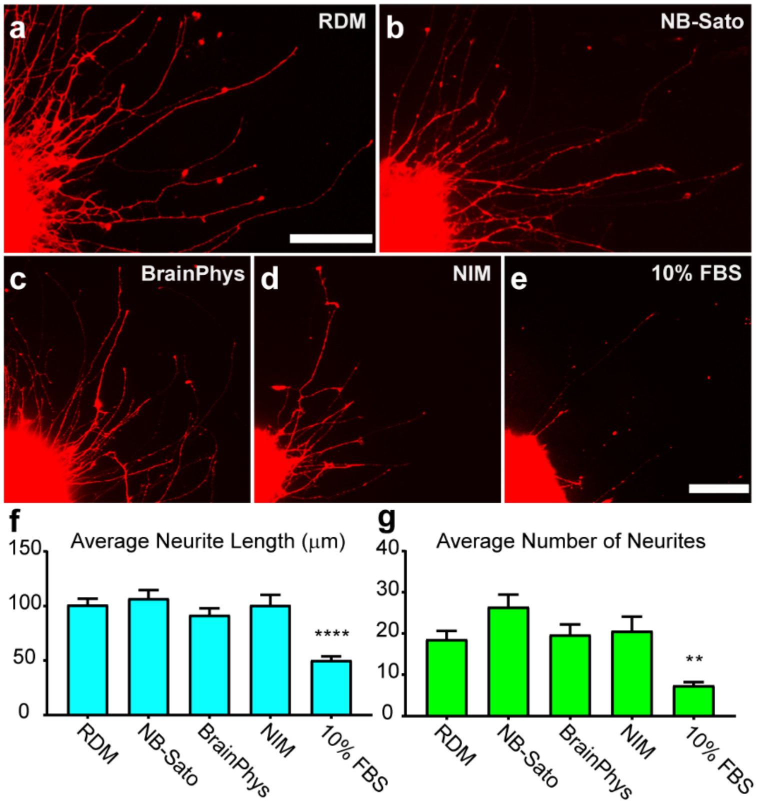

Supplementary Figure S2. Optimizing culture media formulations for enhanced neurite outgrowth. (a-e) mCherry-positive RGCs were analyzed for neurite outgrowth in various culture media including RDM (n=51), NB-Sato (n=56), BrainPhys (n=60), NIM (n=38), and 10% FBS (n=47). (f-g) Results indicated that RDM, NB-Sato, BrainPhys, and NIM were all comparable for average neurite length and average neurite number, with only 10% FBS significantly lower than all others in both length and number. One-way ANOVA was used to compare media, $^{**}p<0.01$, $^{****}p<0.001$. Error bars represent s.e.m. Scale bar: 100 µm.
